# Improvement method for cervical cancer detection: A comparative analysis

**DOI:** 10.32604/or.2022.025897

**Published:** 2022-10-10

**Authors:** NUR AIN ALIAS, WAN AZANI MUSTAFA, MOHD AMINUDIN JAMLOS, AHMED ALKHAYYAT, KHAIRUL SHAKIR AB RAHMAN, RAMI Q. MALIK

**Affiliations:** 1 Faculty of Electrical Engineering & Technology, Universiti Malaysia Perlis, UniCITI Alam Campus, Sungai Chuchuh, Padang Besar, 02100 Perlis, Malaysia; 2 Advanced Computing (AdvCOMP), Centre of Excellence, Universiti Malaysia Perlis (UniMAP), Pauh Putra Campus, Arau, 02600, Perlis, Malaysia; 3 Faculty of Electronic Engineering & Technology, Universiti Malaysia Perlis, UniCITI Alam Campus, Sungai Chuchuh, Padang Besar, 02100, Perlis, Malaysia; 4 Department of Computer Technical Engineering, College of Technical Engineering, The Islamic University, Najaf, 54003, Iraq; 5 Department of Pathology, Hospital Tuanku Fauziah, Kangar, Perlis, 02000, Malaysia; 6 Medical Instrumentation Techniques Engineering Department, Al-Mustaqbal University College, Babylon, Hillah, 51001, Iraq

**Keywords:** Cervical cancer, detection, pap smear, images

## Abstract

Cervical cancer is a prevalent and deadly cancer that affects women all over the world. It affects about 0.5 million women anually and results in over 0.3 million fatalities. Diagnosis of this cancer was previously done manually, which could result in false positives or negatives. The researchers are still contemplating how to detect cervical cancer automatically and how to evaluate Pap smear images. Hence, this paper has reviewed several detection methods from the previous researches that has been done before. This paper reviews pre-processing, detection method framework for nucleus detection, and analysis performance of the method selected. There are four methods based on a reviewed technique from previous studies that have been running through the experimental procedure using Matlab, and the dataset used is established Herlev Dataset. The results show that the highest performance assessment metric values obtain from Method 1: Thresholding and Trace region boundaries in a binary image with the values of precision 1.0, sensitivity 98.77%, specificity 98.76%, accuracy 98.77% and PSNR 25.74% for a single type of cell. Meanwhile, the average values of precision were 0.99, sensitivity 90.71%, specificity 96.55%, accuracy 92.91% and PSNR 16.22%. The experimental results are then compared to the existing methods from previous studies. They show that the improvement method is able to detect the nucleus of the cell with higher performance assessment values. On the other hand, the majority of current approaches can be used with either a single or a large number of cervical cancer smear images. This study might persuade other researchers to recognize the value of some of the existing detection techniques and offer a strong approach for developing and implementing new solutions.

## Introduction

Cervical cancer is one of the primary causes of gynecologic cancer and one of the most common and dangerous diseases for women, even though it can be treated if detected early. Cervical cancer is cancer that forms when cells on the cervix grow abnormally. There is a large volume of published studies utilizing the Pap smear test to detect pre-cancer in the uterine cervix [1,2]. This type of cancer also remains one of the major public health challenges in several countries, especially countries with low and middle income, in terms of the financial aspect and logistical issues [3]. Previous studies have reported that this cancer is the fourth most pervasive cancer type, which affects the life of many people worldwide [4–6]. A large and growing body of literature has investigated the main cause of cervical cancer, stating that the long-lasting infection with a certain type of human papillomavirus (HPV) is passed from one person to another during sex. Non-human papillomavirus-associated adenocarcinomas (NHPVAs) are uncommon uterine cervix tumors with a deceptive appearance [7–9].

HPV will affect at least half of all sexually active persons at some point in their lives, but only a small percentage of women will develop cervical cancer. A pap smear test is used to detect cervical cancer in most cases and is known as a widely used screening procedure for cervical cancer. However, in recent years, practitioners have executed this evaluation manually, and the results are still controversial due to the accuracy of the diagnosis in detecting cervical cancer cells. In addition, the evaluation is done using the naked eye to determine the type of cervical cell. Furthermore, due to human error, this manual screening approach has a high rate of false-positive results [10].

However, far too little attention has been paid to the occurrence of cervical cancer that can be effectively reduced with preventive clinical management strategies, including vaccines and regular screening examinations [11]. It has previously been observed that early diagnosis and classification of cervical lesions greatly boost the chance of successful treatments of patients [12]. The main objective of the initial diagnosis and classification of cervical cancer is to reduce the mortality rate [13,14]. This cancer can be successfully treated with earlier detection. The findings from existing research recognize the critical role played by the screening test in reducing the mortality rate caused by cervical cancer.

In the past years, the Pap smear test has attracted much attention and is best known as a preventive approach used in the current medical field for detecting cervical cancer [15,16]. This test demands a specialized and labor-intensive analysis of cytological preparations to trace potentially malignant cells from both the internal and external cervix surfaces. The cytopathologist analyzes the microscopic fields by screening for abnormal cells. The use of slide digital cytology imaging to increase cytological diagnosis accuracy could be beneficial. Recent evidence suggests that screening diseases, including cervical cancer, breast cancer, and colorectal cancer, using cell images from slide cells has been widely applied in recent years [17–19]. However, poor image quality due to the uneven staining, complex backgrounds and overlapped cell clusters poses a greater challenge in nuclei segmentation [20].

In addition, biomedical signal processing, which entails analyzing, improving, and presenting pictures obtained via x-ray, ultrasound, MRI, and other methods, has the same concept as biomedical image processing. Image processing is a technique for performing operations on an image to improve or extract relevant information. It is one type of signal processing that processes an input of a picture and turns the output maybe into an image or characteristics/features associated with that image. Most recent attention has focused on image processing for classifying cervical cells. However, the nature of the accurate classification of Pap smear images is still in the improvement stage for better performance. It is still one of the challenging tasks in medical image processing, and its performance can be enhanced by extracting and selecting well-defined features and classifiers [21]. Computer-assisted cervical cancer screening based on automated recognition of cervical cells offers the potential to minimize errors and increase the accuracy of the test when compared to manual screening. Traditional approaches rely heavily on cell segmentation accuracy and discriminative hand-crafted feature extraction [22]. The purpose of this paper is to review recent research on automated detection methods available for the classification of cervical cancer.

## Review of Study

Numerous studies have attempted to suggest that image pre-processing may have a dramatic positive effect on the quality of feature extraction and image analysis results [23–26]. For example, Jahan et al. [27] have demonstrated that pre-processing outlines the methods such as cleaning, integration, transformation, and reduction. The main goals of data preparation are to reduce data size, establish data correlations, standardize data, remove outliers, and extract features. Before adopting Machine Learning (ML) models, the basic six steps for coping with the intended dataset must be performed. The process of importing the library, importing the dataset, working with missing data in the dataset, encoding categorical data, and splitting the dataset into training and test sets are all done in a methodical way [27]. A number of studies have found that image segmentation is a common approach used in various pre-processing image applications. Medical imaging, video surveillance, and object detection are some practical applications of image segmentation. The segmentation approach is the method for automatically or semi-automatically extracting the Region of Interest (ROI) from an image [28]. Thus, it enables the suggested method to engage with the image region of interest (ROI) rather than pixels on a grid. After that, the Simple Liner Iterative Clustering (SLIC) output then advances to the second stage, the Density-based Spatial Clustering of Application with noises (DBSCAN) clustering algorithm for similar grouping of super pixels based on their density. DBSCAN produces a clustered image, with each cluster being a nucleus candidate. There are fewer image regions to evaluate at this step, which reduces computing time and prevents a non-nucleus image from being classified as a nucleus. DBSCANs only input parameter is a threshold, which determines to cluster using a density distance function.

In a different study, an artificial intelligence accurate diagnosis solution (AIATBS) is developed to improve cervical liquid-based thin layer cell smear diagnosis according to clinical (The Bethesda System) TBS criteria [29]. The Darknet53 framework was used to coordinate the target detection training, and a YOLOv3 detection model was obtained. Then, integration of XGBoost and a logical decision tree is applied to optimize the parameters provided by the learning process, in which a full cervical liquid-based cytology smear TBS diagnosis system that includes a quality control solution is created. The 121 characteristics from the YOLOv3 detection model, Xception classification model, Patch classification model, and nucleus segmentation model were fed into an XGBoost model for diagnostic model training. Positive and negative squamous intraepithelial lesions were predicted to be positive or negative. A basic XGBoost model for squamous intraepithelial lesions TBS classification was used to further classify the positive results. The system adapts to diverse standards, staining methods, and scanners when it comes to sampling preparation.

An investigation and research finding by Xue et al. [30] also point toward the application of Automatic Visual Evaluation (AVE) to predict pre-cancer based on a digital image of the cervix. This approach has been seen to be a low-cost means of enhancing human performance. However, taking AVE beyond proof-of-concept and into use as a functional complementary tool in visual screening has several challenges. Creating AVE robust across images recorded with several devices is one of them. A new deep learning-based clustering approach is being used to see whether images taken by three different devices (a standard smartphone, a custom smartphone-based handheld device for cervical imaging, and a clinical colposcope with SLR digital camera-based imaging capability) can be distinguished from one another in terms of visual appearance/content within their respective cervix regions. Two established ImageNet pre-trained networks, known as ResNet50 and Vgg16, are used in the study. The representative deep learning classification network is a classification network that has attained excellent performance on the ImageNet dataset to extract features, allowing authors to use the transfer learning technique. The findings and analysis show a need to design a system that reduces the variance between photos acquired from different devices. It also emphasizes the importance of a vast number of training images from various sources for reliable device-independent AVE performance around the world [30].

In addition, Stacked Denoising Autoencoders (SDAEs) are applied to improve the performance of normal Stacked Autoencoders (SAE). However, when examining a larger number of input samples, SDAE’s convergence rate takes longer, given by 2′16, 2′18, and 2′14, s since each sample will be taken into account. The suggested h6, h8, and h4 systems add the Fine-tuned Stacked Denoising Autoencoder (FSDAE), which denoises using a minibatch of samples rather than the entire data from supplied input images. The proposed FSOD-second GAN phase will augment the collected images with segregated classes, related types, and stages to minimize overfitting due to the subsequent detection and classification. Several data augmentation procedures, such as rotation, flip, shift, and zoom, have been used to increase the overall quantity of data. Resizing and cropping the input photographs to a width and height of 100X100 pixels, as well as recoloring the grayscale color channel, were used to enhance and pre-process them. The final image will be a matrix with each row consisting of abnormally flattened grayscale pixels [31].

One of the more significant findings to emerge from this review is that pre-processing of images plays an important phase in image processing techniques, specifically for the detection method of cervical cancer cells. Although the previous researcher has used several techniques, the most common method is augmentation. The augmentation method is one method that is able to increase the cardinality of the training dataset and avoid fitting. Apart from that, this helps in increasing the accuracy of the overall network of the convolutional layer structure. Furthermore, the total number of images can be increased with the application of techniques like rotation, flipping, shifting and zooming for data augmentation.

### Detection method based on cells/pap smear images

Deep learning is a computer-aided diagnostics (CAD) based system investigated widely to classify cervical Pap cells. However, deep learning may provide poor performance for a multiclass classification task when there is an uneven distribution of data which is prevalent in the cervical cell dataset. A study has been conducted by Rahaman et al. [32] to address those limitations by proposing DeepCervix, a hybrid deep feature fusion (HDFF) technique. A hybrid ensemble technique comprising 15 different machine learning algorithms such as random forest, bagging, rotation forest, and J48 is able to perform better than an individual algorithm. Various pre-trained deep learning models in this study, including VGGNet, ResNet, ResNetV2, Inception- Net, InceptionResNetV2, XceptionNet, DenseNet, and NasNet has been trained. Results obtained have indicated that a combination of VGG16, VGG19, ResNet50 and XceptionNet provides the best results for this task [32].

**Figure 1:**
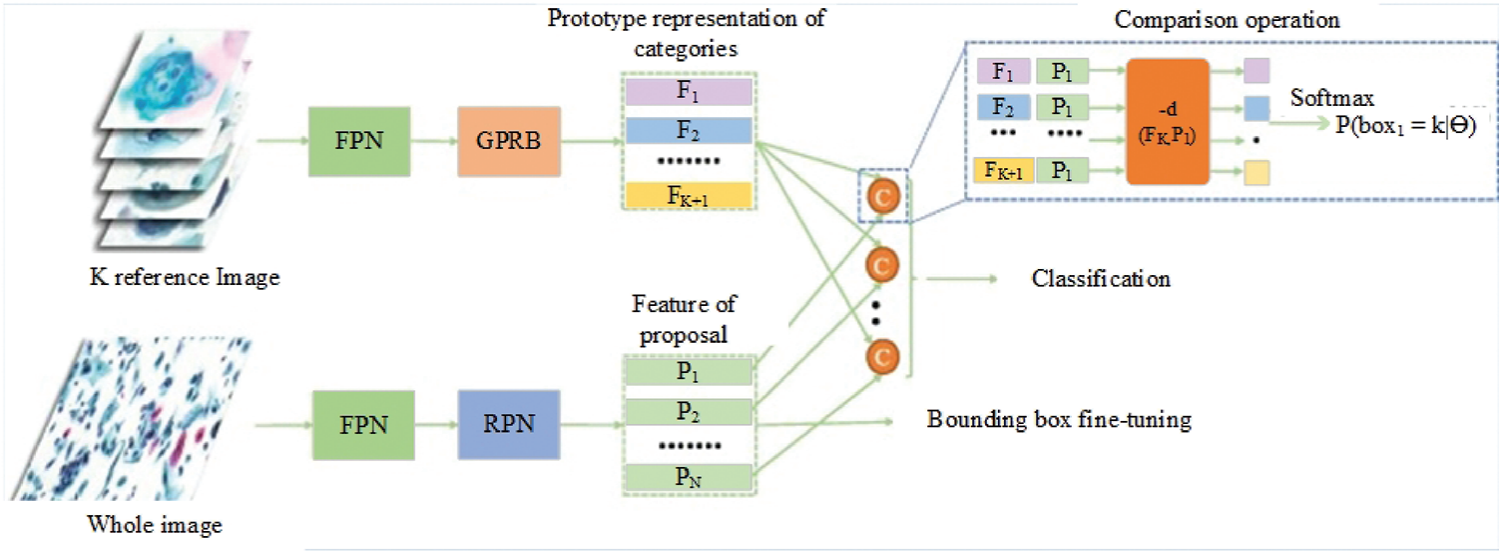
The overall structure of the Comparison detector proposed for feature extraction, an RPN for generating proposals and a head for the proposed classification and bounding box regression [36].

More recent studies have confirmed that current discussions in biomedical technology relate to the method for detecting cervical cancer. There have been several studies in the literature reviews related to the detection of cervical cancer cells with the objective of aiding pathologists. In the year 2021, Cao et al. [33] proposed a method in which they describe a novel deep learning method named attention feature pyramid network (AttFPN) for abnormal cervical cells. The AttPFN method consists of two main components. It comprises an attention module mimicking the way pathologists read a cervical cytology image as well as a multi-scale region-based feature fusion network guided by clinical knowledge to fuse the refined structure for detecting abnormal cervical cells at different scales. The proposed method outperformed the other related deep learning methods of Faster R-CNN with Feature Pyramid Network (FPN), worthy of comparison to experienced pathologists with a 10-year of experience on an independent dataset. The findings are consistent with the study by several researchers, which proposed the utilization of the Faster R-CNN method for the detection of cervical cancer cells [34,35]. Besides, Tang et al. [36] proposed the comparison detector based on a proposal-based detection framework which often consists of a backbone network for feature extraction, an RPN for generating proposals and a head for the proposed classification and bounding box regression. The overall structure of the comparison detector proposed is shown in Fig. 1.

The research study by Chen et al. [37] has proposed a study that focuses on improving the accuracy of cervical cell classification by considering resource limitations. A compact and effective model that meets design requirements for embedded devices is built with a lightweight convolutional neural network (CNN) architecture to create a highly efficient model with fewer parameters and calculations. The proposed method’s basic steps are as follows:

- Prepare the image samples by pre-processing the datasets.
- On the target dataset, use transfer learning to train different teacher models. First, download the already trained CNNs, then fine-tune CNNs to the target dataset. Then, based on the training results, determine the final teacher model.
- From the final instructor model, get the soft labels.
- On the target dataset, train the lightweight student CNN models using dark knowledge loss, cross-entropy loss, as well as soft and hard labels.
- Only use the traditional cross-entropy loss and hard labels to test the lightweight models on the target dataset [37].

A Multi-Task Network (MTN) is one of the methods proposed in a study based on Y-Net’s architecture and performs two tasks: nuclear segmentation and classification. The network’s segmentation component features an encoder-decoder structure. The fundamental feature extraction activities in the encoder are handled by efficient spatial pyramid (ESP) modules. The decoder receives the encoder’s final feature representation and constructs a nuclear mask with the same spatial resolution as the input using up sampling and pyramid spatial pooling (PSP) modules. Information can be shared between the encoder and the decoder by concatenating skip links from the encoder to the decoder. The diagnostic component of the MTN is made up of more ESP modules, which lead to an average global pooling module and two completely connected layers. A single convolution conducts down sampling processes, halving the spatial resolution of the feature maps. Bilinear interpolation is used for up sampling. After each down sampling, up sampling, ESP, and PSP module, batch normalization and ReLU activation are implemented. The modules that make up the MTN and the learning via proxy labels are described in the following sections [38].

In a different study, Diniz et al. [39] has shown an efficient ensemble to classify the segmented regions (nucleus candidate) returned from the pre-processing phase. The ensemble method consists of the Decision Tree (DT), Nearest Centroid (NC) and k-Nearest Neighbors (k-NN). The findings of the study show that this ensemble method achieved the best result concerning the F1 and recall values. With the same objective, three segmentation strategies for automated segmentation of cervical cell nuclei in the presence of debris are described by Arya et al. [40]. Automated Seed Region Growing, Extended Edge Based Detection, and Modified Moving Segmentation is three segmentation approaches. Extraction of the nuclei of cervical cells, k-means approaches are presented. Using the morphological trait of a nucleus, these techniques extract the area of nuclei from smear images. Some debris has an area that matches the nucleus of normal cells, which can cause interference and false-positive results. This study describes three strategies for automated segmentation of cervical cell nuclei in the presence of debris. It comprises Automated Seed Region Growing, Extended Edge Based Detection, and Modified Moving Segmentation approaches. These techniques extract the area of nuclei from smear images using the morphological attribute of the nucleus. Some debris has a nucleus that matches regular cells, causing interference and false-positive results. Research demonstrates that Modified Moving k-means are more accurate in identifying dysplastic in the presence of debris [40].

A workload-reducing algorithm for analysis of cell nuclei features from Pap smear images. An investigation has been done with the involvement of eight traditional machine learning methods to perform a hierarchical classification [41]. The classifier involved were: AdaBoost, Decision Tree (DT), Gaussian Naive-Bayes (GNB), k-Nearest Neighbors (k-NN), Multi-Layer Perceptron (MLP), Nearest Centroid (NC), Random Forest (RF), and Ridge. A hierarchical classification methodology is one method developed for computer-aided screening of cell lesions with an aim to provide another side of view from the Pap smear images based on the nuclei detection of cervical cancer. The methodology starts with the extraction of features from each nucleus segmented in the database. Region Props, Haralick’s features, Local Binary Patterns (LBP), Threshold Adjacency Statistics (TAS), Zernike moments, and Gray Level Co-occurrence Matrix were among the algorithms used (GLCM). All the programmes were written in Python, with Region Props and GLCM coming from the scikit-image package and the rest from the Mahotas package. Morphological and other features are also included in the study. The study found that hierarchical classification provided better findings than those without it. In 2021, Pirovano et al. [42] explained in a study how to apply the suggested method (classifier with regression constraint) to the novel task of categorizing tiles from cytology images in the context of cervical cancer. In that paper, with the application of an attribution strategy, a demonstration has been made to the model learned to discover the cells responsible for the anticipated label under weak supervision. The three suggested architecture (Resnet-101 classifier, Resnet-101 Regressor and Resnet-101{Classifier + Regressor}) surpasses a simple classifier and other state-of-the-art approaches for ordinal classification in terms of overall accuracy and severity prediction. Furthermore, the suggested method is successfully tuned to achieve a higher sensitivity as a tool that can help practitioners [42].

Recently, a convolutional neural network-based detector has been used to lessen the reliance on hand-crafted features and eliminate the need for segmentation. These strategies, on the other hand, tend to produce an excessive number of false-positive predictions. Therefore, to resolve this issue, a global context-aware framework was created with the use of an image-level classification branch and a weighted loss to incorporate global context information. A global context-aware network with soft-scale anchor matching (SSAM) is proposed to optimize the parameters. This method involves a backbone network, image-level classification branch (ILCB) and cervical cell detection branch. This branch’s prediction is paired with cell detection to filter out erroneous positive predictions. The backbone network provides shared features for image-level categorization and cervical cell detection. DarkNet is used as the backbone network. Abnormal cervical image existence is catered to using the application of ILCB, which is directly attached to the top of the backbone network. The cervical cell detection branch consists of a three-level FPN, and the detection head attached to each feature level of FPN is used to anticipate where cervical cells will be spotted and which class they will belong to. Table 1 will illustrate the summary of past studies related to nucleus detection.

**Table 1:** Summary results of prevailing works for nucleus detection of the cervical cancer cell

Authors	Title	Result and advantages
Jia et al. [43]	Detection of cervical cancer cells in a complex situation based on an improved YOLOv3 network	MAP of 78.87%, 8.02%, 8.22% and 4.83% higher than SSD (Single Shot Multi-Box Detector), YOLOv3 (You Only Look Once) and ResNet50.
Ali et al. [44]	Machine learning-based statistical analysis for early-stage detection of cervical cancer	A Random Tree (RT) accuracy biopsy (98.33%), cytology (98.65%)
		Random Forest (RF) and Instance-Based K-nearest neighbor (IBk) provided the best performance for Hinselmann (99.16%) and Schiller (98.58%), respectively.
Zhang et al. [45]	Quantitative detection of cervical cancer based on time series information from smear images	Accuracy 98.3%
		Sensitivity 98.1%
		Specificity 97.9%.
Chitra et al. [46]	An optimized deep learning model using a Mutation-based Atom Search Optimization algorithm for cervical cancer detection	Accuracy 98.38%
		Sensitivity 98.83%
		Specificity 98.5%.
		Precision 98.58%
		Recall 99.3%
		F-score 98.25%
Cao et al. [33]	A novel attention-guided convolutional network for the detection of abnormal cervical cells in cervical cancer screening	Online Database
		Sensitivity = 95.83%
		Specificity = 94.81%
		Accuracy = 95.08%
		AUC = 0.991
		External Dataset (110 cases and 35,013 images)
		Sensitivity = 91.30%
		Specificity = 90.62%
		Accuracy = 90.91%
		AUC = 0.934
		Diagnostic time is 0.04s/image compare to average time of pathologist 14.83s/image.
Devi, et al. [47]	Cervical Cancer Classification from Pap Smear Images Using Modified Fuzzy C Means, PCA, and KNN	Minimum accuracy 94.15%, Maximum accuracy 96.28%, Average accuracy 94.86%, Sensitivity 97.96%,
		Specificity 83.65%
		F1-score 96.87%,
		Precision 96.31%
Bhatt et al. [48]	Cervical cancer detection in pap smear whole slide images using convNet with transfer learning and progressive resizing	Accuracy (99.70%)
		Precision (99.70%)
		Recall (99.72%)
		F-Beta (99.63%)
		Kappa scores (99.31%)
Desiani et al. [49]	Bi-path Architecture of CNN Segmentation and Classification Method for Cervical Cancer Disorders Based on Pap-smear Images	Accuracy = 90%
		Sensitivity (SN)
		Specificity (SP)
		F1-score


## Methodology

### Dataset

Herlev is a widely used dataset, and this image database has been used to design the detection technique. In addition, most researchers used the Herlev University image datasets to improve the design and development process. Herlev Pap database is compiled by Herlev University Hospital (Denmark) and the Technical University of Denmark. The database contains 917 pictures manually sorted into groups by professional cytotechnicians and physicians. Surface squamous, intermediate squamous, columnar, mild dysplasia, moderate dysplasia, extreme dysplasia, and *in situ* cancer are among the seven cervical cell classifications in the database. In addition, various cell and nucleus properties are extracted [2].

In this study, 105 pap smear images were used. The database falls under the category of NiSIS or Nature inspired Smart Information System (EU coordination action, contract 13569), with a particular focus on the group “Nature-Inspired Data Technology”. The data is accessible over the internet (http://mde-lab.aegean.gr/index.php/downloads). Table 2 provides the details of the dataset used for the nucleus detection method. Seven types of cells fall under the category of normal cells and abnormal cells. The normal cells consist of normal superficial, normal intermediate and normal columnar types. In contrast, the abnormal cells consist of mild dysplastic, moderate dysplastic, severe dysplastic and carcinoma in situ type of cells. The total numbers of images in this dataset are 917.

**Table 2:** Descriptions of seven-classes cells from the Herlev (single cells) dataset

Class	Type	Number of cells
Normal cells	Normal superficial	74
	Normal intermediate	70
	Normal columnar	98
Abnormal cells	Mild dysplastic	182
	Moderate dysplastic	146
	Severe dysplastic	197
	Carcinoma in situ	150
	Total	**917**

### Experimental procedure

The experiment is done based on several approaches used by previous researchers in past studies. This study considers the method for nucleus detection for cervical cells based on pap smear test images. The dataset used is the established Herlev dataset. The image is processed based on the suggested approach for improving existing segmentation techniques such as thresholding, trace region boundary, contrast enhancement, edge detection, as well as a morphological and watershed approach using Matlab R2021a. The processed image is then compared to the ground truth using image quality assessment for performance analysis. Finally, the values calculated are compared to determine the better performance approach for nucleus detection.

### Performance analysis

Performance analysis is a series of heterogeneous computer-aided tools that assess a system’s performance at several levels of abstraction, making the task more difficult. The performance of the analysis process can be enhanced through the extraction of a common object model. Five regularly used performance metrics from the literature were used in the reviewed studies. Accuracy, precision, recall, geometric mean and F1-score are the performance measurements. These performance measures were calculated using mathematical equations [31,50–52]. Details of the performance metric as per shown in Table 3.

**Table 3:** Performance assessment metrics [41]

Metric	Equation	Goal
Precision (Prec.)	$\frac{TP}{TP+FP}$	Indicate, among the positive ratings, the correct amount calculated
Recall (Rec.)	$\frac{TP}{TP+FN}$	The correct detection of the abnormal nuclei
F1-score (F1)	$2\times \frac{precision\times recall}{precision+recall}$	The harmonic means of precision and recall
Accuracy (Acc.)	$\frac{TP+TN}{TP+FP+TN+FN}$	Compute the percentage of correct tests (TP and TN) across all of the results.
Specificity (Spec.)	$\frac{TN}{TN+FP}$	Determines whether the approach appropriately excludes nuclei without lesions.
*	TP = True positive	
	TN = True negative	
	FP = False positive	
	FN = False negative	

Note: *The numbers of correctly predicted positive and negative classes are TP and TN, respectively, while the numbers of wrongly predicted positive and negative classes are FP and FN.

Furthermore, Jia et al. [53] showed that true positives (TP), false positives (FP), true negatives (TN), and false negatives (FN) are used to create indicators in the confusion matrix. Accuracy, precision, sensitivity, specificity, F-Index, and negative predictive value (NPV) are common measures in biomedical segmentation. Precision is often used in conjunction with sensitivity and refers to the ratio of correctly splatted foreground pixels. The ratio of pixels in ground truth that match the separated ones is referred to as sensitivity.

The average harmonic value of precision and sensitivity is known as the F-Index. The NPV is a metric for how comprehensive a set of results is. Other metrics, such as the Dice coefficient and the Jaccard Index, provide a more comprehensive assessment of segmentation. The extracted contours are estimated fairly using Volumetric Similarity (VS). Visual accuracy (VA) is a visual evaluation of segmentation. The following is a list of the metrics referenced in [53] that lead to false-positive results. The histogram of an image in image processing usually refers to a histogram of pixel intensity values.

### Qualitative results

One of the most broadly utilized performance analysis methods is qualitative analysis. Probabilistic statements about the algorithm’s performance and weaknesses are based on human visual perception [40,54].

In a study by Arya et al. [40], the first step in analyzing the results of three segmentation techniques to predict the dysplastic in cervical cells in the presence of debris is extracting the Region of interest (ROI). Normal cell nuclei, abnormal cell nuclei, and debris are detected in ROI, and the area of all the objects is computed. Some debris has a region that matches the nucleus of normal cells, which could interfere with the outcome and lead to false-positive results. The histogram of an image in image processing usually refers to a histogram of pixel intensity values.

### Quantitative analysis

Quantitative analysis is a numerical-based way of obtaining information on an algorithm’s performance without involving any human interaction. Smear photos have a lot of debris in the background, and the nucleus and cytoplasm are in the foreground. The number of items, precision, sensitivity, F-measure, specificity, accuracy and PSNR are calculated in a complicated context. The calculated values can be calculated based on the segmented images. These findings demonstrate the importance of validating image quality using the suggested techniques on the Pap smear dataset [40].

## Results

A reviewed method based on several segmentation techniques has been tested using Matlab. Table 4 has tabulated the processed images for different types of cervical cells using four reviewed methods. Methods used are (1) Edge detection and morphological approach, (2) Watershed Approach, (3) Thresholding and trace region boundaries in the binary image and (4) Enhance Grayscale Images using Contrast Enhancement Technique. Nuclei are displayed in the final image for normal cells in the segmented images, while a blank image is formed for aberrant cells. Overall, according to the qualitative analysis, the thresholding and trace region boundaries in binary images outperform the other traditional algorithms in terms of segmentation performance, regardless of the number of objects employed [40]. Several methods have been introduced for cervical cancer detection in the area of nucleus detection. In this study, the image database is processed based on the four methods reviewed and was written as a new image. Table 4 shows that method 1 shows favorable results compared to the other methods. However, the other methods are also able to detect nuclei but are limited to certain types of cells. Performance analysis is a series of heterogeneous computer-aided tools

**Table 4:** The processed image based on the enhanced method

Type of cells	Normal columnar	Normal intermediate	Normal superficial	Mild dysplastic	Moderate dysplastic	Severe dysplastic	Carcinoma *in situ*
Benchmark	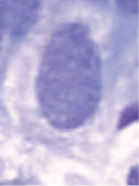	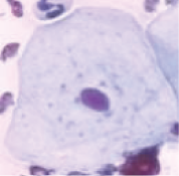	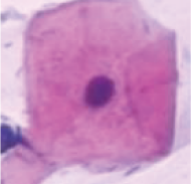	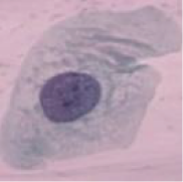	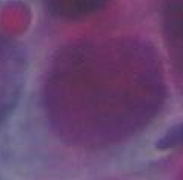	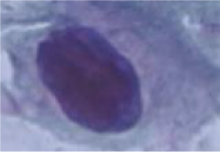	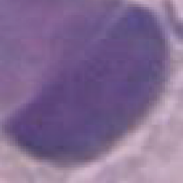
Method 1: Thresholding and Trace region boundaries in the binary image	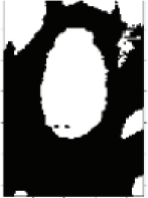	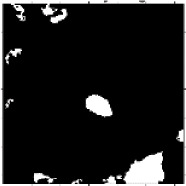	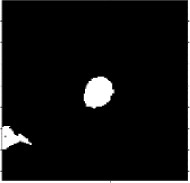	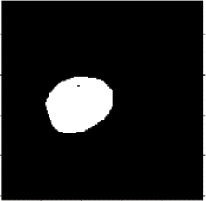	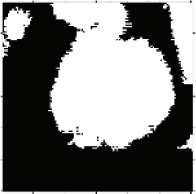	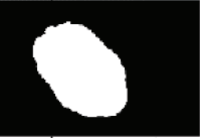	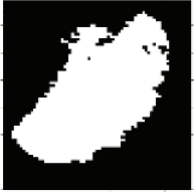
Method 2: Enhance Grayscale Images using Contrast Enhancement Technique	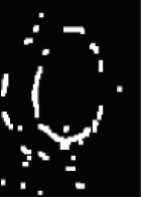	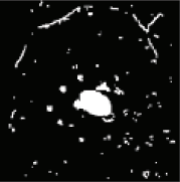	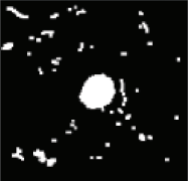	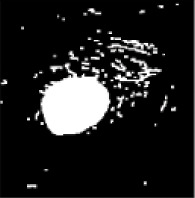	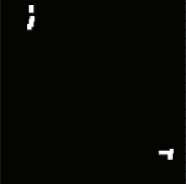	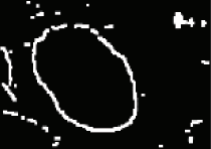	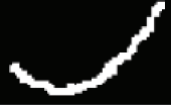
Method 3: Edge Detection and Morphological Approach	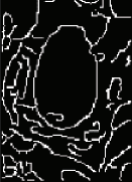	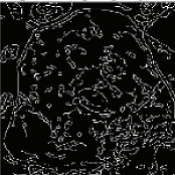	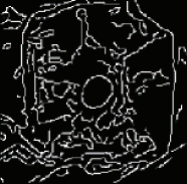	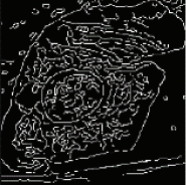	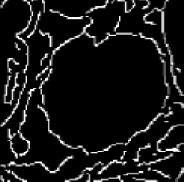	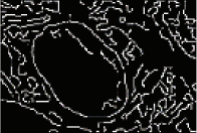	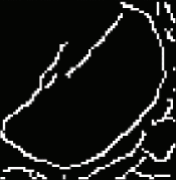
Method 4: Watershed Approach	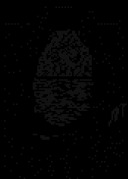	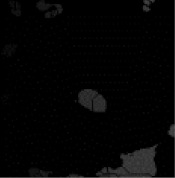	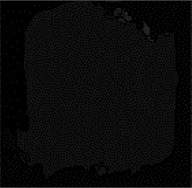	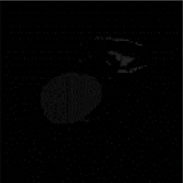	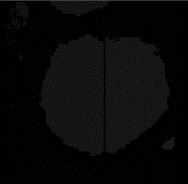	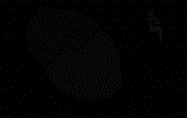	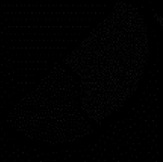

Table 5 until Table 8 has demonstrated the calculated values of precision, sensitivity, f-measure, specificity, accuracy and PSNR for each approach tested. Based on the result shown in Table 5, the approach of thresholding and tracing region boundaries in binary mage showed a favorable result in the ability to detect the nucleus of the cervical cancer cells for all different types of cells. This approach is able to detect all nuclei from seven types of cells with a high value of precision, sensitivity, f-measure, specificity, accuracy and PSNR. The highest values obtained are for a severe dysplastic cell which shows the consistent highest values of precision 1, sensitivity 98.77%, F-measure 99.37%, specificity 98.41%, accuracy 98.77% and PSNR 25.74%. Thus, the tabulated data has proved this method is able to perform a good image pre-processing for the nucleus detection of a cervical cancer cell.

**Table 5:** Qualitative results based on precision, sensitivity, F-measure, specificity, accuracy and PSNR for Method 1: Thresholding and Trace region boundaries in the binary image

Type of cells	Precision	Sensitivity	Specificity	Accuracy	PSNR
Normal columnar	0.96	78.41	93.06	83.46	9.21
Normal intermediate	0.98	85.62	96.70	89.77	14.57
Normal superficial	0.99	87.32	98.45	92.70	12.44
Mild dysplastic	0.99	89.59	94.80	90.72	12.44
Moderate dysplastic	1.00	96.93	98.76	96.90	18.93
Severe dysplastic	1.00	98.77	98.41	98.77	25.74
Carcinoma in situ	0.99	98.32	95.66	98.04	20.20
**Average**	**0.99**	**90.71**	**96.55**	**92.91**	**16.22**

The calculated data of precision, sensitivity, F-measure, specificity, accuracy and PSNR has been recorded in Table 6 for method 2: Enhance grayscale images using contrast enhancement technique. This approach has fluctuating values for all calculated values. Values of sensitivity, F-measure and accuracy are in the high range of around 50% to 100%. The PSNR value might not be the highest compared to method 1. However, the values are still more than 3% and up to 14.80%. This method yields high values for accuracy of 95.41% and 94.07% for the cell type of moderate dysplastic and severe dysplastic, respectively.

**Table 6:** Qualitative results based on precision, sensitivity, F-measure, specificity, accuracy and PSNR for Method 2: Enhance grayscale images using contrast enhancement technique

Type of cells	Precision	Sensitivity	Specificity	Accuracy	PSNR
Normal columnar	0.69	96.19	5.39	66.89	4.92
Normal intermediate	0.67	94.31	2.86	64.82	4.58
Normal superficial	0.51	96.59	0.82	49.92	3.00
Mild dysplastic	0.80	94.71	7.38	77.04	6.40
Moderate dysplastic	0.98	95.82	69.92	94.07	12.48
Severe dysplastic	1.00	95.33	99.95	95.41	14.80
Carcinoma in situ	0.94	95.25	39.21	89.51	10.19
**Average**	**0.80**	**95.46**	**32.22**	**76.81**	**8.05**

The accuracy value is quite high and in the range of other existing techniques reviewed.

Next, for method 3: Edge detection and morphological approach, the calculated data of precision, sensitivity, F-measure, specificity, accuracy and PSNR have been recorded in Table 7. This approach has fluctuated values for all calculated values, which possess quite a similar pattern to Method 2. Values of sensitivity, F-measure and accuracy are in the high range of around 50% to 100%. The PSNR value might not be the highest compared to Method 1. However, the values are still more than 3% and up to 14.39%. This method yields high values for accuracy of 96.31%, 94.98% and 90.52% for cell types of severe dysplastic, moderate dysplastic and carcinoma *in situ*, respectively. The value of accuracy is quite high and in the range of other existing techniques reviewed. This method is a better option compared to Method 2 but still a less likely option compared to Method 1.

**Table 7:** Qualitative results based on precision, sensitivity, F-measure, specificity, accuracy and PSNR for Method 3: Edge detection and morphological approach

Type of cells	Precision	Sensitivity	Specificity	Accuracy	PSNR
Normal columnar	0.69	97.00	1.73	67.33	5.02
Normal intermediate	0.67	96.54	0.74	65.88	4.75
Normal superficial	0.51	96.35	1.84	50.58	3.06
Mild dysplastic	0.80	97.66	2.17	78.35	6.65
Moderate dysplastic	0.97	97.97	0.62	94.98	13.41
Severe dysplastic	0.99	97.68	1.68	96.31	14.39
Carcinoma in situ	0.91	98.11	4.34	90.51	9.83
**Average**	**0.79**	**97.33**	**1.87**	**77.71**	**8.16**

Lastly, for Method 4: watershed approach, although this method has resulted in the lowest values of all calculated data as in Table 8, it still promotes an opportunity to be improved for better performance. Therefore, compared to the other proposed algorithms, Thresholding and trace region boundaries in binary images outperform them all. The related qualitative analysis demonstrates good image segmentation ability [40]. Based on the values calculated, it is easier to choose a better option for image pre-processing in detecting the nucleus of cervical cancer cells.

**Table 8:** Qualitative results based on precision, sensitivity, F-measure, specificity, accuracy and PSNR for Method 4: Watershed approach

Type of cells	Precision	Sensitivity	Specificity	Accuracy	PSNR
Normal columnar	0.63	4.74	93.66	32.29	1.73
Normal intermediate	0.66	5.89	93.99	33.86	1.82
Normal superficial	0.24	2.78	91.41	45.76	2.66
Mild dysplastic	0.56	3.22	92.42	21.24	1.04
Moderate dysplastic	0.96	6.85	92.11	9.38	0.44
Severe dysplastic	0.98	7.42	93.13	9.39	0.43
Carcinoma in situ	0.88	2.77	96.53	11.38	0.53
**Average**	**0.70**	**4.81**	**93.32**	**23.33**	**1.24**

### Discussion

The tested approach for cervical cell nucleus detection has greatly improved compared to the literature for method 1: Thresholding and trace region boundaries in the binary images. The highest values for this method are 1.00, 98.77, 98.76,98.77 and 25.74 for precision, sensitivity, specificity, accuracy and PSNR values, respectively. Based on the previous studies that has been reviewed, the performance of approaches used previously has resulted in precision values of 0.98 [46], 0.96 [47], 0.99 [48], sensitivity 98.83% [46], 95.83% [33], 97.96% [47], specificity 97.9% [45], 98.5% [46], 90.62% [33], 83.65% [47], 99.70 [48], accuracy 98.3% [45], 98.38 [46], 95.08% [33], 96.28% [47], 99.70% [48], 90% [49]. The values obtained from the experiment for method 1, which is thresholding and trace region boundaries in a binary image, outperform other methods in terms of precision, sensitivity and specificity from previous studies. This shows that this method can provide good data for further classification of the cervical cancer cell type.

## Conclusion

Several approaches and analysis methods are studied and reviewed in this paper that has been developed to create an end-to-end framework for cervical cancer diagnosis and classification. All of the strategies proposed have been designed to work with multivariate datasets. The recommended methods have also been expanded to include determining the kind and stage of cervical cancer in addition to diagnosing it. Experiments were conducted at the training, validation, and testing stages. The results show that the highest performance assessment metric values obtain from Method 1: Thresholding and Trace region boundaries in a binary image with the highest values of precision 1.0, sensitivity 98.77%, specificity 98.76%, accuracy 98.77% and PSNR 25.74% for a certain type of cell. Meanwhile, for average, the values of precision were 0.99, sensitivity 90.71%, F-measure 94.00%, specificity 96.55%, accuracy 92.91% and PSNR 16.22%. The experimental results are then compared to the existing methods from previous studies. They show that the improvement method is able to detect the nucleus of the cell with higher performance assessment values in sensitivity, specificity and precision. According to the publications reviewed, the current techniques have shortcomings, resulting in poorer classification accuracy in specific cell types. Most existing techniques, on the other hand, work on single or many cervical cancers smear images. Furthermore, there is little evidence that these algorithms will work in clinical situations. Furthermore, there seems to be no evidence that these algorithms will perform in clinical settings in developing countries (where 85% of cervical cancer cases occur) since competent cytologists and funds to purchase commercial segmentation software are limited. In conclusion, this research may motivate other field researchers to recognize the potential of some of the methodologies investigated, as well as give a solid platform for creating and implementing new ways.

## Data Availability

Data Availability Statement: The dataset analyzed during the current study was derived from The Herlev Database, which consists of 917 manually isolated pap smear cells. Dataset is available on the corresponding website http://mde-lab.aegean.gr/index.php/downloads (accessed on 15 March 2022).
